# Two further patients with Warsaw breakage syndrome. Is a mild phenotype possible?

**DOI:** 10.1002/mgg3.639

**Published:** 2019-03-28

**Authors:** Roberta Bottega, Luisa M. R. Napolitano, Anna Carbone, Enrico Cappelli, Fabio Corsolini, Silvia Onesti, Anna Savoia, Paolo Gasparini, Flavio Faletra

**Affiliations:** ^1^ Institute for Maternal and Child Health – IRCCS “Burlo Garofolo” Trieste Italy; ^2^ Structural Biology Laboratory Elettra‐Sincrotrone Trieste S.C.p.A. Trieste Italy; ^3^ Medical Genetics Unit Città della Salute e della Scienza University Hospital Turin Italy; ^4^ Clinical and Experimental Hematology Unit “G. Gaslini” Children’s Hospital Genoa Italy; ^5^ U.O.S.D. Centro di Diagnostica Genetica e Biochimica delle Malattie Metaboliche “G. Gaslini” Children’s Hospital Genoa Italy; ^6^ Department of Medical Science University of Trieste Trieste Italy

**Keywords:** DDX11, mutations, Warsaw Breakage Syndrome

## Abstract

**Background:**

Warsaw Breakage Syndrome (WABS) is an ultra rare cohesinopathy caused by biallelic mutation of *DDX11* gene. It is clinically characterized by pre and postnatal growth delay, microcephaly, hearing loss with cochlear hypoplasia, skin color abnormalities, and dysmorphisms.

**Methods:**

Mutational screening and functional analyses (protein expression and 3D‐modeling) were performed in order to investigate the presence and pathogenicity of DDX11 variant identified in our patients.

**Results:**

We report the clinical history of two sisters affected by WABS with a pathological mytomicin C test carrying compound heterozygous mutations (c.2507T > C / c.907_920del) of the *DDX11* gene. The pathogenicity of this variant was confirmed in the light of a bioinformatic study and protein three‐dimensional modeling, as well as expression analysis.

**Conclusion:**

These findings further extend the clinical and molecular knowledge about the WABS showing a possible mild phenotype without major malformations or intellectual disability.

## 1 INTRODUCTION

Warsaw Breakage Syndrome (WABS; OMIM #613398) is an ultra rare genetic disease caused by biallelic mutations of the *DDX11 *(DEAD/H box‐11) gene. DDX11 (also named Ch1R1) belongs to Rad3/XPD FeS helicase family, whose members unwind DNA with a 5′ to 3′ directionality(Bharti et al., 2014). Sharing sequence similarity with FANCJ, XPD, and RTEL‐1 helicases, DDX11 is involved in the maintenance of genomic stability and cohesion of sister chromatids(Sun et al., 2015). WABS is characterized by prenatal and postnatal growth delay with microcephaly, various degrees of intellectual disability, hearing loss with cochlear malformation, and skin pigmentation abnormalities. Dysmorphic features include sloping forehead and narrow bifrontal diameter, prominent eyes, upslanting palpebral fissures, epicanthus, hypoplastic alae nasi, and small mouth (Alkhunaizi et al., 2018; Bailey, Fryer, & Greenslade, 2015; Capo‐Chichi et al., 2013; Eppley, Hopkin, Mendelsohn, & Slavotinek, 2017; van der Lelij et al., 2010). Patients might also present hypotonia, cardiac malformations, and bone (radial and fibula) malformations.

Cells from WABS patients show an increase in spontaneous and mitomycin C (MMC)‐induced chromosomal breakage and sister chromatid cohesion defects with a typical railroad chromosome structures, showing cytogenetic overlap with Fanconi anemia (FA) and Roberts breakage syndrome (RBS) (Capo‐Chichi et al., 2013; van der Lelij et al., 2010). Herein, we report the clinical history of two sisters with WABS, who are compound heterozygous for two novel mutations of DDX11.

## 2 MATERIALS AND METHODS

### 2.1 DNA sequencing

Written informed consent was obtained from patients or from relatives/guardians whenever applicable. All experiments were carried out in accordance with the approved guidelines. Genomic DNA was extracted from peripheral blood of both patients and parents. Sanger sequencing was performed in order to confirm mutation and for segregation study, as previously described. Primers used for the amplification are available upon request. PCR products were bidirectionally sequenced using an ABI 3100 automated sequencer (Applied Biosystem).

### 2.2 Cell culture and mitomycin C survival assay

Patient's and wild type lymphoblast cell lines (LFB) were generated from primary lymphocytes isolated from peripheral blood as described in Ravera et al. (2016). Cells were grown at 37°C in RPMI supplemented with 10% FBS and antibiotics. Mytomycin C (MMC) survival assay were performed using a standard method. Briefly, lymphoblast cells were collected and exposed to increasing concentrations of MMC (0–333 nmol/L) for 5 days. Then, cells were resuspended in PBS plus 0.05% BSA and 0.5 μg/ml propidium iodide for 10 min at 4°C. Cell viability was analyzed by flow cytometry.

### 2.3 Protein expression analyses

Fractionated (nucleus/cytoplasm) and not‐fractionated cell extracts were prepared from LFB cell lines using M‐PER™ Mammalian Protein Extraction Reagent (Thermo Fisher Scientific) as previously reported (Bottega et al., 2018). Primary antibodies were used as follows: anti‐DDX11 (Santa Cruz, sc‐271711, 1:500) and anti‐β‐actin (Santa Cruz, sc‐47778, 1:2000). Immuno‐reactivity was visualized using the Enhanced Chemiluminescent SuperSignal™ West Femto Maximum Sensitivity Substrate (Pierce).

### 2.4 Bioinformatic analyses

The effect of the missense variant was evaluated by means of several prediction programs, such as Combined Annotation Dependent Depletion (CADD; http://cadd.gs.washington.edu/home), Mutation Taster (http://www.mutationtaster.org/), Mutation Assessor (http://mutationassessor.org/), and PolyPhen‐2 (http://genetics.bwh.harvard.edu/pph2/). Multiple sequence alignments were generated with the Clustal Omega server (Sievers et al., 2011) and manually modified to account for the position of the secondary structure elements. The following groups of residues were considered very similar and highlighted in yellow in Figure 3a: Glu/Asp, Arg/Lys, Val/Ile/Leu/Met/Cys, Gly/Ala, Tyr/Phe/Trp, Ser/Thr/Pro, Asn/Gln. A three‐dimensional model was generated using the RaptorX Structure Prediction program (Källberg et al., 2012). The best template was found to be the 10 Å resolution CryoEM structure of human XPD, as found in the TFIIH complex (PDB ID: 5IVW, chain W) with a *p*‐value 7.4e‐12. Although this complex was not determined at atomic resolution, the structure of XPD was built on the basis of homologous archaeal structures determined at high resolution. The picture of the DDX11 atomic model (Figure 3b) was generated using PyMOL (http://pymol.org/).

## 3 RESULTS

**Figure 1:**
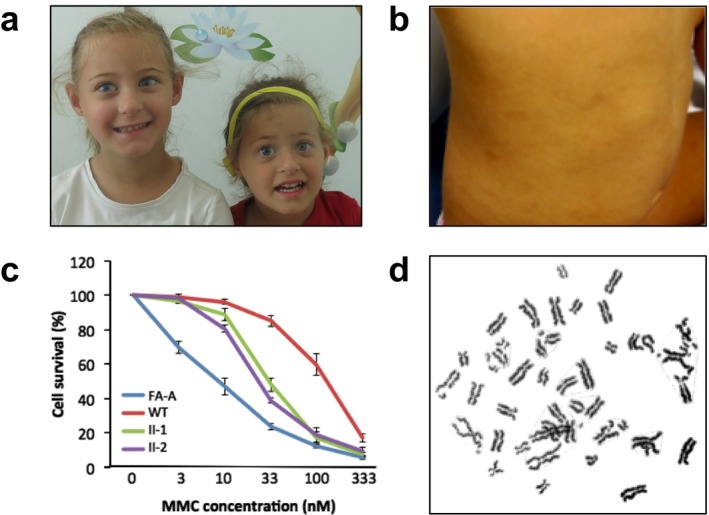
Clinical and cellular features. (a) Typical dysmorphisms observed in Warsaw Breakage Syndrome patients: bilateral epicanthal folds, upslanting palpebral fissures, prominent nose and columella with hypoplastic alae nasi, small ears, and micrognathia. (b) Trunk image showing the café‐au‐lait spots. (c) Comparison of the mitomycin C (MMC) induced cell survival analysis in LFB cells from a wild‐type (WT), patients carrying the *DDX1* mutations (II‐1 and II‐2) and a Fanconi anemia patient carrying mutations in *FANCA* gene (FA‐A). WBS patients shows an intermediate phenotype. (d) Karyotype analyses after MMC induction

Two 5‐year‐old and 4‐year‐old sisters, born of nonconsanguineous healthy parents were referred to the Genetic Department because of hearing impairment. Both sisters had intra‐uterine growth retardation (IUGR); at birth (37 gs) they were small for gestational age with weight, length, and head circumference of 2,230 g, 44.5 cm, 30.5 cm and 1,820 g, 42 cm, 28 cm, respectively. During the dysmorphological evaluation the anthropometric values were under the 3° centile with a more pronounced microcephaly (−3.34 and −4.09 *SD*). They both showed sloping forehead with apparently narrow bifrontal diameter, prominent eyes with bilateral epicanthal folds, upslanting palpebral fissures, prominent nose and columella with hypoplastic alae nasi, small ears, and micro/retrognathia. In the trunk and legs skin they showed several café‐au‐lait spots (Figure 1a,b). Computed tomography of brain and magnetic resonance imaging revealed a rather small and rounded cochlea. Both patients present a hearing loss that appears moderate (Pure tone audiometry: wave V elicitable down to 80 dB on the right ear and 70 dB on the left) in the older sister and profound (Pure tone audiometry: wave V undetectable) in the younger one. The IQ tests performed with the Wppsi‐III scale showed a normal global cognitive IQ (92) with a homogeneous pattern (Verbal IQ: 100; Performance: 93) in the older sister and a borderline global cognitive IQ of 75 (Verbal IQ: 69; Performance: 93) in the younger sister, which mostly affects her verbal ability, also considering the severe hearing loss of course.

The hand radiography revealed a mild bilateral shortening of the first metacarpal bone in the young sister as already reported for WABS patients (Eppley et al., 2017). Because of these features, a clinical WABS suspicion was hypothesized. As WABS cells are reported to show MMC sensitivity (van der Lelij et al., 2010), an MMC survival test was performed in lymphoblastoid cells from the two sisters comparing them to FA patient cells (compound heterozygous for c.3660del and c.50dup of the *FANCA* gene). This revealed an intermediate sensitivity (Figure 1c) (Bottega et al., 2018). The karyotype analysis after MMC induction showed the typical railroad chromosomes (Figure 1d), suggesting a diagnosis of WABS.

**Figure 2:**
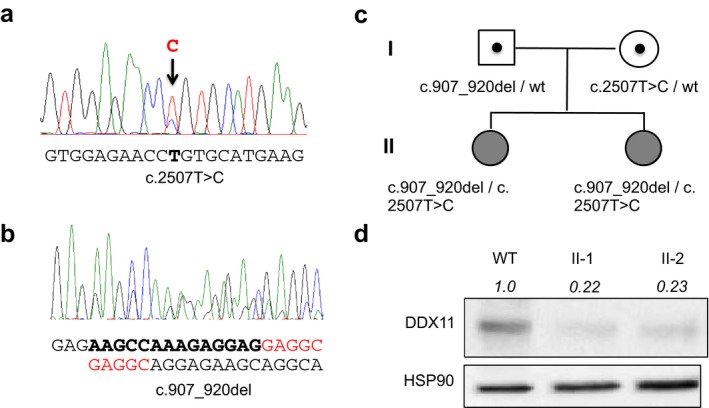
Patients’ mutations and DDX protein expression. (a) Sanger sequencing validation of c.2507T > C (p.Leu836Pro) and (b) c.907_920del (p.Lys303Glufs*22) DDX11 mutations. (c) Pedigree of the family. (d) Western blot analyses of WBS patients (II‐1 and II‐2) showing a partial expression (22% and 23%, respectively) of DDX protein compared to wild type. HSP90 was used as loading control and normalizator for protein quantification

We therefore screened for mutations the *DDX11* gene by Sanger sequencing and detected two variants (c.2507T > C; p.Leu836Pro and c.907_920del; p.Lys303Glufs*22) in both sisters (Figure 2a,b). Segregation analyses demonstrated the paternal and maternal origin of the missense variant and deletion, respectively (Figure 2c). Both variants are not reported in pathogenicity and healthy control databases. Whereas the deletion of 14 nucleotides represents a clear deleterious variant that leads to frameshift and premature stop codon, the outcome of a substitution is generally unclear; p.Leu836Pro was thus investigated in order to determine its effect. It was predicted to be likely pathogenic according to the ACMG/AMP guidelines and by all the pathogenicity prediction tools used (MutationTaster, score = 0.999, “Disease causing”; MutationAssessor, score = 3.6, “High”; PolyPhen‐2, score = 0.995, “Probably damaging”) except CADD (score = 17, “Neutral”).

**Figure 3:**
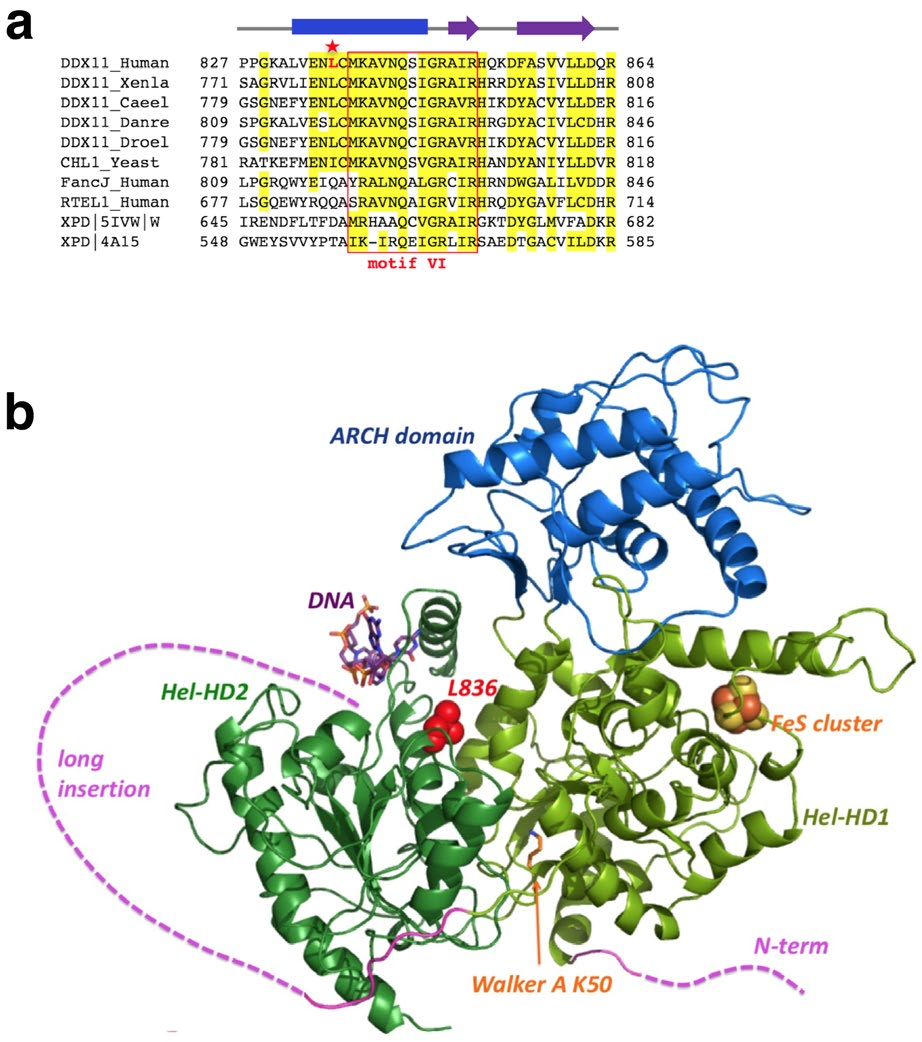
DDX11 alignment and 3D structure. (a) Multiple sequence alignment of eukaryotic DDX11 with the other human FeS cluster helicases. Residues conserved in most DDX11 sequences are highlighted in yellow. The mutated L836 residue is shown in red and indicated by a star. The predicted secondary structure is shown on the top (rectangular blue bar for α‐helix and purple arrows for β‐strands). (b) A DDX11 atomic model has been generated starting from XPD crystal structure (PDB ID: 4A15) with the ARCH domain in blue, HD1 and HD2 domains in two shades of green and the FeS cluster in orange. Unstructured regions/insertions in DDX11 are indicated in purple. The position of L836 is shown at the interface between the domains

Moreover, multiple sequence alignment among DDX11 proteins and other FeS helicases show that the position of Leu836 is conserved among orthologs (Figure 3a).

Residue Leu836, in the context of the FeS helicases framework, is located within the HD2 catalytic domain, just before the helicase motif VI, that is relatively well conserved among different species (Figure 3a) (Sievers et al., 2011). Secondary structure predictions suggest that Leu836 is imbedded in an α‐helix structure that is not compatible with a proline substitution (Källberg et al., 2012). The Leu836Pro substitution would cause a significant disruption in the protein structure (Figure 3b). Moreover, template‐based modeling of human DDX11 suggests that this residue would sit in a critical position, at the interface between the *h*omology *d*omain 1 and 2 (HD1, HD2) and ARCH domains, and possibly close to the DNA binding region (Figure 3b) (Kuper, Wolski, Michels, & Kisker, 2012).

Finally, the DDX11 protein expression was investigated by western blot (Figure 1c) demonstrating a very low expression (22%) compared to the wild‐type (100%). This residual protein expression is more likely caused by the allele carrying the c.2507T > C, as the p.Lys303Glufs*22 mutant form derived from the c.907_920del allele is not detectable and is probably degraded.

## 4 DISCUSSION

WABS is a recently described disease reported for the first time in 2010 by van der Lelij et al. (2010). It belongs to the group of cohesinopathies, which include WABS, Roberts syndromes (RBS), and Cornelia de Lange Syndrome (CdLS). WABS is considered a cohesinopathy since *DDX11 *mutation are associated with premature sister chromatid separation (Banerji, Skibbens, & Iovine, 2017). WABS patients show a clinical overlap with RBS and CdLS, even if these two last conditions are characterized by a more severe phenotype including several malformations such as cochlear, palate, limb, heart, kidney, genital, and gastrointestinal defects. The clinical picture of our two patients is similar to that previously described for other WABS, except for the cognitive development delay and the metacarpal bone defects. Indeed, even though all the cases described so far show an intellectual disability associated with language impairment (Alkhunaizi et al., 2018), the intellectual performance of the two sisters was “normal” in the older and “borderline” in the younger, suggesting that, at least at present, their phenotype is mild. Nevertheless, considering the microcephaly and the young age, patients will be followed up to evaluate their performances.

Of note, abnormalities of the first metacarpal bone are infrequent and inconstant features in WABS families as well as in patients from the same family, as seen in our two patients.

Regarding the MMC breakage test, its use as a diagnostic tool for WABS is controversial as the cytogenetic defects were not observed in all patients (Alkhunaizi et al., 2018; Bailey et al., 2015; Capo‐Chichi et al., 2013; Eppley et al., 2017; van der Lelij et al., 2010). Although the MMC breakage sensitivity was critical to diagnose our family, further investigation is needed to characterize the chromosomal instability in WABS. However, it is necessary to emphasize that chromosomal instability assays are difficult to perform and not always reproducible among different laboratories.

The diagnostic suspicion was confirmed by the molecular diagnosis that allowed us to identify two novel variants of *DDX11 *(c.907_920del and c.2507T > C). Whereas the first one was considered deleterious leading to a premature stop codon (p. p.Lys303Glufs*22), we considered the second one (p.Leu836Pro) as pathogenic because it: (i) affects a highly conserved amino acid among DDX11 orthologous; (ii) should cause a significant change in the protein structure; (iii) hits a critical residue located at the interface between the HD1, HD2, and ARCH domains which may be close to the DNA binding region; (iv) is associated with low DDX11 expression level in patients’ LFB, suggesting that the mutant protein is partially degraded or has a reduced half life. Consistently with our data, all missense mutations of DDX11 tested for their in‐vitro expression are expressed even if at low level (Alkhunaizi et al., 2018; Capo‐Chichi et al., 2013). Considering that the knockout of Ddx11 mice resulted in embryonic lethality at E10.5 (Inoue et al., 2007 Jul), taken together, these data lead us to hypothesize that a complete loss‐of‐function of DDX11 gene is incompatible with life. As a consequence, only patients carrying at least one hypomorphic mutation in DDX11 exhibit the disease. Indeed, in most of the WABS families described so far (7 out of 9) at least one allele could be regarded as potentially nondeleterious (missense/in‐frame 3nt deletion), since it is compatible with the production of a rather stable hypomorphic protein.

The exceptions consist in one family described by Alkhunaizi et al. (2018 ^) ^in which the homozygous c.2638dupG is located in the C‐term of the protein, which could retain some functional activity, and in another family described in Bailey et al. (2015 ^) ^whose splice site mutation (c.638 + 1G>A) in intron 5 was not investigated for a possible in‐frame product.

WABS is considered an ultra rare condition since, until now, only 14 patients from nine unrelated families are reported. In very rare autosomal recessive diseases the prevalence increases in consanguineous families with a consequent homozygous state of mutations. In WABS, only four families out of nine carry a homozygous mutation suggesting that this syndrome could be more frequent than expected and recalling the need to report new cases for its better characterization.

## CONFLICT OF INTEREST

The authors declare no conflict of interest.
